# d-Phenyl­glycinium bromide

**DOI:** 10.1107/S1600536813004807

**Published:** 2013-03-02

**Authors:** Mohanadoss Parthasarathy, Kannan Arun Kumar, Rengasamy Gopalakrishnan

**Affiliations:** a Crystal Research Laboratory, Department of Physics, Anna University, Chennai 600 025, India; b Department of Chemistry, Loyola College (Autonomous), Chennai 600 034, India

## Abstract

In the crystal of the title salt, C_8_H_10_NO_2_^+^·Br^−^, the bromide anions and the phenylglycinium cations are ­linked through N—H⋯Br, O—H⋯Br and C—H⋯O hydrogen bonds, generating sheets lying parallel to (001).

## Related literature

For a similar compound with a different halogen anion, see: Ravichandran *et al.* (1998 ▶). For related structures and background, see: Srinivasan *et al.* (2001 ▶); Bouchouit *et al.* (2004 ▶); Ramaswamy *et al.* (2001 ▶); Bouacida *et al.* (2006 ▶); Thomsen *et al. *(1994 ▶). For biological importance, see: Satyam *et al.* (1996 ▶); Jayasinghe *et al.* (1994 ▶); Chun *et al.* (2010 ▶); Thomas & West (2011 ▶).
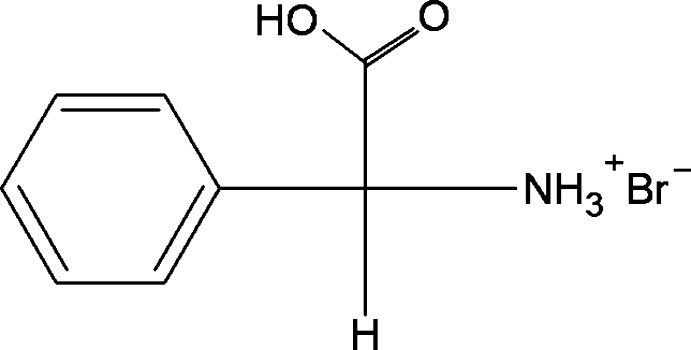


## Experimental

### 

#### Crystal data

C_8_H_10_NO_2_^+^·Br^−^
*M* *_r_* = 232.08
Orthorhombic, 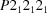

*a* = 5.5240 (5) Å
*b* = 7.4735 (5) Å
*c* = 23.1229 (18) Å
*V* = 954.60 (13) Å^3^
*Z* = 4
Mo *K*α radiation
μ = 4.27 mm^−1^
*T* = 295 K
0.35 × 0.30 × 0.25 mm

#### Data collection

Bruker Kappa APEXII CCD diffractometer
Absorption correction: multi-scan (*SADABS*; Bruker, 2004 ▶) *T*_min_ = 0.317, *T*_max_ = 0.415
5824 measured reflections
2170 independent reflections
2003 reflections with *I* > 2σ(*I*)
*R*_int_ = 0.022

#### Refinement

*R*[*F* ^2^ > 2σ(*F* ^2^)] = 0.022
*wR*(*F* ^2^) = 0.046
*S* = 1.03
2170 reflections
114 parameters
H-atom parameters constrained
Δρ_max_ = 0.25 e Å^−3^
Δρ_min_ = −0.29 e Å^−3^
Absolute structure: Flack (1983 ▶)
Flack parameter: 0.011 (8)

### 

Data collection: *APEX2* (Bruker, 2004 ▶); cell refinement: *APEX2* and *SAINT* (Bruker, 2004 ▶); data reduction: *SAINT* and *XPREP* (Bruker, 2004 ▶); program(s) used to solve structure: *SIR92* (Altomare *et al.*, 1993 ▶); program(s) used to refine structure: *SHELXL97* (Sheldrick, 2008 ▶); molecular graphics: *ORTEP-3 for Windows* (Farrugia, 2012 ▶) and *Mercury* (Macrae *et al.*, 2008 ▶); software used to prepare material for publication: *PLATON* (Spek, 2009 ▶).

## Supplementary Material

Crystal structure: contains datablock(s) I, global. DOI: 10.1107/S1600536813004807/pk2465sup1.cif

Structure factors: contains datablock(s) I. DOI: 10.1107/S1600536813004807/pk2465Isup2.hkl

Supplementary material file. DOI: 10.1107/S1600536813004807/pk2465Isup3.cml

Additional supplementary materials:  crystallographic information; 3D view; checkCIF report

## Figures and Tables

**Table 1:** Hydrogen-bond geometry (Å, °)

*D*—H⋯*A*	*D*—H	H⋯*A*	*D*⋯*A*	*D*—H⋯*A*
N1—H1*B*⋯Br1^i^	0.89	2.54	3.3586 (17)	154
N1—H1*C*⋯Br1^ii^	0.89	2.57	3.429 (2)	163
N1—H1*A*⋯Br1	0.89	2.45	3.3166 (18)	164
O1—H1*D*⋯Br1^iii^	0.82	2.39	3.2027 (17)	171
C7—H7⋯O2^iv^	0.98	2.59	3.527 (3)	159

Symmetry codes: (i) 
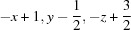
; (ii) 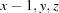; (iii) 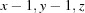; (iv) 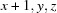.

**Fig. 1.**
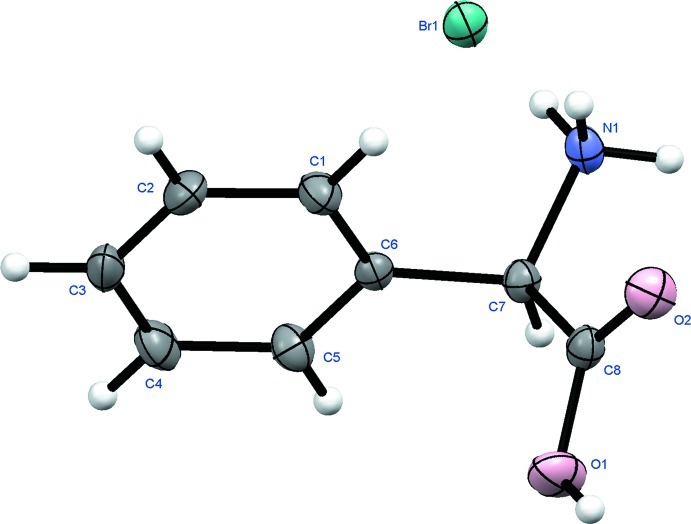
Displacement ellipsoid plot of the molecular structure drawn at the 40% probability level.

**Fig. 2.**
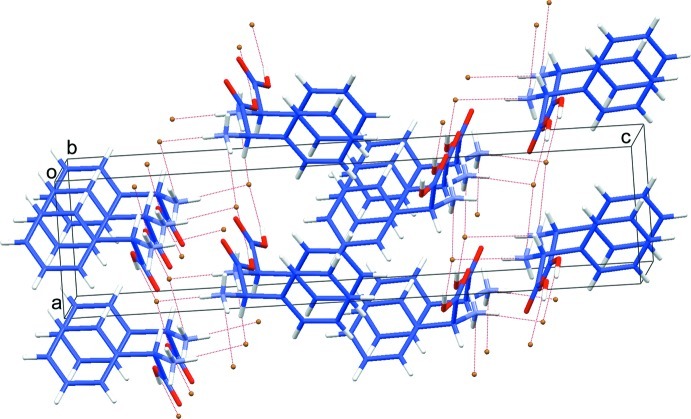
Part of the crystal structure showing the two dimensional anionic-cationic (0 0 1) sheet formed through N—H···Br, O—H···Br and C—H···O interactions viewed down c axis.

## supplementary crystallographic information

### Comment

D-Phenylglycine is an important constituent in the production of semisynthetic penicillins and cephalosporins. Recently the usages of some phenylglycine derivatives in the synthesis of antitumor drugs and other pharmacological applications have been found to be increasing (Satyam et al., 1996; Jayasinghe et al., 1994). Phenylglycine has been reported as a delivery tool for improving l-dopa absorption (Chun et al., 2010) and also found to have anti-inflammatory activity (Thomas et al., 2011). The torsion angle N1-C7-C8-O1, which indicates the relative orientation of the carboxyl group and the amino N atom, is 15.5 (3)° and close to the corresponding value of 18.9°(5) reported for D-Phenylglycine Hydrochloride (Ravichandran et al., 1998). The orientation of the phenyl ring as described by the torsion angle C5—C6—C7—N1 is 130.05 (3)°. The intermolecular interaction between the molecular ions are primarly decided by hydrogen bonding. The hydrogen bonds N1—H1A···Br1, N1—H1B···Br1^i^ [Symmetry code: (i) -x+1, y-1/2, -z+3/2], N1—H1C···Br1^ii^ [Symmetry code: (ii) x-1, y, z] and O1—H1D···Br1^iii^ [Symmetry code: (iii) x-1, y-1, z] and C7—H7···O2^iv^ [Symmetry code: (iv) x-1, y-1, z] hydrogen bond interconnects the molecular ions to form an extensive two-dimensional molecular sheet parallel to (001) plane. Parallel stacking of these sheets along [0 0 1] direction constitute the molecular packing of the crystal.

### Experimental

The title compound (I), was prepared by mixing a 1:1 ratio of *D*-Phenylglycine and hydrobromic acid in water solvent. The suitable single-crystal of the compound was selected for X-ray analysis from the above solution by slow evaporation method.

### Refinement

The hydrogen atoms associated with C atoms were identified from the difference electron density peaks and subsequently treated as riding atoms with distances of d(C–H) = 0.98 Å (for CH) with *U*_iso_(H) = -1.5*U*_eq_(C) and d(C–H) = 0.93 Å (for aromatic CH) with *U*_iso_(H) = 1.2*U*_eq_(C). The carboxylic acid hydrogen was constrained to a distance of d(O–H) = 0.82 Å with *U*_iso_(H) = 1.5*U*_eq_(C) and the positions of NH_3_ H atoms were also treated as riding about the parent atom.

### Crystal data

**Table:**

C_8_H_10_NO_2_^+^·Br^−^	*F*(000) = 464
*M**_r_* = 232.08	*D*_x_ = 1.615 Mg m^−^^3^
Orthorhombic, *P*2_1_2_1_2_1_	Mo *K*α radiation, λ = 0.71073 Å
Hall symbol: P 2ac 2ab	Cell parameters from 3239 reflections
*a* = 5.5240 (5) Å	θ = 2.6–28.8°
*b* = 7.4735 (5) Å	µ = 4.27 mm^−^^1^
*c* = 23.1229 (18) Å	*T* = 295 K
*V* = 954.60 (13) Å^3^	Block, colourless
*Z* = 4	0.35 × 0.30 × 0.25 mm

### Data collection

**Table:**

Bruker Kappa APEXII CCD diffractometer	2170 independent reflections
Radiation source: fine-focus sealed tube	2003 reflections with *I* > 2σ(*I*)
Graphite monochromator	*R*_int_ = 0.022
ω and φ scan	θ_max_ = 27.5°, θ_min_ = 3.3°
Absorption correction: multi-scan (*SADABS*; Bruker, 2004)	*h* = −6→7
*T*_min_ = 0.317, *T*_max_ = 0.415	*k* = −9→5
5824 measured reflections	*l* = −28→30

### Refinement

**Table:**

Refinement on *F*^2^	Hydrogen site location: inferred from neighbouring sites
Least-squares matrix: full	H-atom parameters constrained
*R*[*F*^2^ > 2σ(*F*^2^)] = 0.022	*w* = 1/[σ^2^(*F*_o_^2^)]
*wR*(*F*^2^) = 0.046	(Δ/σ)_max_ = 0.003
*S* = 1.03	Δρ_max_ = 0.25 e Å^−^^3^
2170 reflections	Δρ_min_ = −0.29 e Å^−^^3^
114 parameters	Extinction correction: *SHELXL97* (Sheldrick, 2008), Fc^*^=kFc[1+0.001xFc^2^λ^3^/sin(2θ)]^-1/4^
0 restraints	Extinction coefficient: 0.0530 (13)
Primary atom site location: structure-invariant direct methods	Absolute structure: Flack (1983)
Secondary atom site location: difference Fourier map	Flack parameter: 0.011 (8)

### Special details

**Table:**

Geometry. All e.s.d.'s (except the e.s.d. in the dihedral angle between two l.s. planes) are estimated using the full covariance matrix. The cell e.s.d.'s are taken into account individually in the estimation of e.s.d.'s in distances, angles and torsion angles; correlations between e.s.d.'s in cell parameters are only used when they are defined by crystal symmetry. An approximate (isotropic) treatment of cell e.s.d.'s is used for estimating e.s.d.'s involving l.s. planes.
Refinement. Refinement of *F*^2^ against ALL reflections. The weighted *R*-factor *wR* and goodness of fit *S* are based on *F*^2^, conventional *R*-factors *R* are based on *F*, with *F* set to zero for negative *F*^2^. The threshold expression of *F*^2^ > σ(*F*^2^) is used only for calculating *R*-factors(gt) *etc*. and is not relevant to the choice of reflections for refinement. *R*-factors based on *F*^2^ are statistically about twice as large as those based on *F*, and *R*- factors based on ALL data will be even larger.

### Fractional atomic coordinates and isotropic or equivalent isotropic displacement parameters (Å^2^)

**Table:**

	*x*	*y*	*z*	*U*_iso_*/*U*_eq_	
C1	0.1060 (5)	0.5444 (3)	0.56980 (9)	0.0370 (5)	
H1	−0.0251	0.5884	0.5906	0.044*	
C2	0.1209 (5)	0.5734 (3)	0.51081 (9)	0.0418 (6)	
H2	−0.0006	0.6372	0.4921	0.050*	
C3	0.3119 (5)	0.5092 (3)	0.47991 (9)	0.0424 (6)	
H3	0.3196	0.5290	0.4402	0.051*	
C4	0.4922 (5)	0.4160 (3)	0.50678 (9)	0.0443 (6)	
H4	0.6229	0.3734	0.4855	0.053*	
C5	0.4804 (4)	0.3849 (3)	0.56595 (9)	0.0355 (5)	
H5	0.6024	0.3204	0.5842	0.043*	
C6	0.2866 (4)	0.4500 (2)	0.59770 (7)	0.0264 (4)	
C7	0.2695 (4)	0.4057 (2)	0.66152 (7)	0.0282 (5)	
H7	0.4217	0.3499	0.6738	0.034*	
C8	0.0650 (4)	0.2773 (3)	0.67328 (8)	0.0311 (5)	
N1	0.2266 (4)	0.5687 (2)	0.69772 (6)	0.0345 (4)	
H1A	0.3369	0.6514	0.6892	0.063 (8)*	
H1B	0.2379	0.5399	0.7350	0.063 (8)*	
H1C	0.0794	0.6117	0.6905	0.047 (7)*	
O1	0.1199 (4)	0.1163 (2)	0.65347 (8)	0.0561 (5)	
H1D	0.0051	0.0486	0.6586	0.084*	
O2	−0.1206 (3)	0.3164 (2)	0.69632 (6)	0.0441 (4)	
Br1	0.71415 (4)	0.82278 (3)	0.681529 (8)	0.03843 (9)	

### Atomic displacement parameters (Å^2^)

**Table:**

	*U*^11^	*U*^22^	*U*^33^	*U*^12^	*U*^13^	*U*^23^
C1	0.0381 (14)	0.0388 (11)	0.0339 (11)	0.0062 (12)	0.0048 (10)	−0.0032 (10)
C2	0.0552 (17)	0.0342 (12)	0.0360 (12)	0.0066 (11)	−0.0030 (11)	0.0072 (10)
C3	0.0638 (18)	0.0364 (12)	0.0270 (10)	−0.0101 (13)	0.0087 (11)	0.0010 (9)
C4	0.0417 (16)	0.0496 (14)	0.0415 (14)	−0.0021 (12)	0.0170 (11)	−0.0082 (11)
C5	0.0277 (13)	0.0429 (12)	0.0360 (12)	0.0004 (11)	0.0035 (9)	−0.0027 (10)
C6	0.0261 (11)	0.0258 (9)	0.0273 (9)	−0.0062 (10)	0.0011 (9)	−0.0013 (7)
C7	0.0261 (13)	0.0316 (10)	0.0269 (9)	−0.0034 (10)	0.0012 (8)	0.0002 (7)
C8	0.0352 (14)	0.0323 (11)	0.0258 (11)	−0.0071 (10)	−0.0018 (9)	0.0014 (8)
N1	0.0377 (13)	0.0396 (9)	0.0262 (9)	−0.0137 (10)	0.0029 (8)	−0.0036 (7)
O1	0.0634 (15)	0.0333 (8)	0.0715 (12)	−0.0161 (9)	0.0238 (10)	−0.0057 (8)
O2	0.0328 (9)	0.0448 (9)	0.0547 (9)	−0.0106 (9)	0.0085 (7)	−0.0021 (8)
Br1	0.04061 (14)	0.03701 (12)	0.03766 (12)	−0.01269 (10)	0.00446 (10)	−0.00279 (9)

### Geometric parameters (Å, º)

**Table:**

C1—C6	1.382 (3)	C6—C7	1.515 (2)
C1—C2	1.383 (3)	C7—N1	1.497 (2)
C1—H1	0.9300	C7—C8	1.507 (3)
C2—C3	1.361 (3)	C7—H7	0.9800
C2—H2	0.9300	C8—O2	1.192 (3)
C3—C4	1.365 (3)	C8—O1	1.323 (3)
C3—H3	0.9300	N1—H1A	0.8900
C4—C5	1.389 (3)	N1—H1B	0.8900
C4—H4	0.9300	N1—H1C	0.8900
C5—C6	1.386 (3)	O1—H1D	0.8200
C5—H5	0.9300		
			
C6—C1—C2	119.8 (2)	C5—C6—C7	119.15 (19)
C6—C1—H1	120.1	N1—C7—C8	107.37 (17)
C2—C1—H1	120.1	N1—C7—C6	112.12 (15)
C3—C2—C1	120.6 (2)	C8—C7—C6	111.19 (16)
C3—C2—H2	119.7	N1—C7—H7	108.7
C1—C2—H2	119.7	C8—C7—H7	108.7
C2—C3—C4	120.4 (2)	C6—C7—H7	108.7
C2—C3—H3	119.8	O2—C8—O1	125.1 (2)
C4—C3—H3	119.8	O2—C8—C7	124.73 (19)
C3—C4—C5	120.0 (2)	O1—C8—C7	110.16 (19)
C3—C4—H4	120.0	C7—N1—H1A	109.5
C5—C4—H4	120.0	C7—N1—H1B	109.5
C6—C5—C4	119.9 (2)	H1A—N1—H1B	109.5
C6—C5—H5	120.0	C7—N1—H1C	109.5
C4—C5—H5	120.0	H1A—N1—H1C	109.5
C1—C6—C5	119.30 (19)	H1B—N1—H1C	109.5
C1—C6—C7	121.4 (2)	C8—O1—H1D	109.5
			
C6—C1—C2—C3	0.2 (4)	C1—C6—C7—N1	−54.0 (3)
C1—C2—C3—C4	−0.3 (4)	C5—C6—C7—N1	130.1 (2)
C2—C3—C4—C5	0.5 (4)	C1—C6—C7—C8	66.2 (2)
C3—C4—C5—C6	−0.6 (3)	C5—C6—C7—C8	−109.7 (2)
C2—C1—C6—C5	−0.2 (3)	N1—C7—C8—O2	15.6 (3)
C2—C1—C6—C7	−176.2 (2)	C6—C7—C8—O2	−107.4 (2)
C4—C5—C6—C1	0.5 (3)	N1—C7—C8—O1	−165.48 (17)
C4—C5—C6—C7	176.5 (2)	C6—C7—C8—O1	71.5 (2)

### Hydrogen-bond geometry (Å, º)

**Table:**

*D*—H···*A*	*D*—H	H···*A*	*D*···*A*	*D*—H···*A*
N1—H1*B*···Br1^i^	0.89	2.54	3.3586 (17)	154
N1—H1*C*···Br1^ii^	0.89	2.57	3.429 (2)	163
N1—H1*A*···Br1	0.89	2.45	3.3166 (18)	164
O1—H1*D*···Br1^iii^	0.82	2.39	3.2027 (17)	171
C7—H7···O2^iv^	0.98	2.59	3.527 (3)	159

Symmetry codes: (i) −*x*+1, *y*−1/2, −*z*+3/2; (ii) *x*−1, *y*, *z*; (iii) *x*−1, *y*−1, *z*; (iv) *x*+1, *y*, *z*.
